# Predictors of Weight Bias in Exercise Science Students and Fitness Professionals: A Scoping Review

**DOI:** 10.1155/2021/5597452

**Published:** 2021-07-05

**Authors:** Lara Zaroubi, Tiffany Samaan, Angela S. Alberga

**Affiliations:** Department of Health, Kinesiology & Applied Physiology, Concordia University, Montreal, QC, Canada

## Abstract

**Background:**

Although previous studies have reported weight bias among students and professionals in exercise science, physical education, kinesiology, and fitness instruction, predictors of weight bias in these professions have not been extensively reviewed.

**Aim:**

The purpose of this scoping review was to explore the available literature on predictors of weight bias in exercise science students and fitness professionals to identify key concepts and research gaps.

**Methods:**

PubMed and ERIC were searched from January 1990 to May 2019. Eighteen studies were included in this review. A thematic analysis was conducted. *Findings*. Six main themes were drawn from these studies including beliefs in the personal controllability of weight; sex differences; enrollment in a health sciences-related program; psychosocial and personal factors; knowledge of obesity; lack of personal history, family, or friend with obesity. Our scoping review highlighted diverse predictors of weight bias among exercise science students and professionals that warrant further study and intervention.

## 1. Introduction

Weight bias is defined as negative beliefs and attitudes toward people living with overweight or obesity [1]. Beliefs and opinions that occur in a conscious and expressive manner are defined as explicit forms of weight bias [2]. Previous studies have documented the existence of explicit weight bias in various settings such as in healthcare among trainees (i.e., nursing, dietetic, and medical students) [2], education (i.e., schoolteachers) [3], and medicine and public health [4–8]. Healthcare providers often associate people with obesity with negative labels and stereotypes such as “lazy”, “weak”, “lack willpower”, “unattractive”, or “unintelligent” [9, 10]. In fact, patients with obesity have reported low trust, poor communication, lack of training, and disrespectful treatment from their healthcare providers [11]. Experiencing weight bias in healthcare settings is particularly harmful because it can negatively affect patient engagement and utilization of healthcare services [11]. Future health professionals' biases are problematic as they may deter both clients and patients from adopting healthy lifestyle choices [12, 13]. For example, individuals with obesity experiencing explicit weight bias can experience physical and emotional tribulations including stress, anxiety, depression, avoidance or lower motivation for exercise, and disordered eating [14] and may not be receiving appropriate care for their health conditions [15].

While there is a lack of clearly defined approaches to reduce weight bias among healthcare professionals, a systematic review of weight bias reduction interventions identified preprofessional educational training in healthcare programs as one potential target [16]. Since many healthcare professionals and health educators working with adults and children with obesity often have educational training backgrounds in physical education, health, and kinesiology, it would be important to understand predictors of weight bias among students and professionals in these fields. Numerous studies have reported weight bias among students in physical education [17], kinesiology, and exercise science programs [18, 19] and professionals in these fields (e.g., physical education teachers, fitness instructors) [20, 21]. Weight bias has been observed among physical education teachers whereby they have expressed lower expectations in performance and abilities of students with obesity compared to their normal weight peers [20]. Students enrolled in physical education programs who have not addressed their own weight-biased attitudes throughout their training program may express these biases as future physical education teachers toward their own students with obesity [20]. In fact, physical educators' weight bias, lower expectations, and experiences of weight teasing of children with obesity may lead to poorer self-esteem, body image issues, lower physical activity performance and lower motivation if students experience differential treatment because of their body weight [22–24]. There is evidence that suggests physical education students display greater weight bias toward the third year of their program compared to their first year and display higher weight bias compared to other health science students [17]. This further highlights the importance of understanding factors associated with weight bias in the early formative years to avoid the propagation of weight bias in professional practice and its potential to negatively affect the treatment and quality of healthcare of individuals with obesity.

While it is clear that weight bias is pervasive in students and professionals in the field of exercise science, we need a better understanding of the factors that predict weight bias in this field to avoid the propagation of weight bias and its negative consequences on patient adherence, health behaviors, and clinical outcomes. The purpose of this scoping review was to synthesize all available literature pertaining to the predictors of weight bias in students and professionals in exercise sciences. For the purpose of this paper, students in exercise science refer to students enrolled in undergraduate or graduate programs in exercise science, physical education, and kinesiology and professionals in exercise science include physical educators, fitness instructors, exercise physiologists, and exercise specialists. We used a scoping review methodology due to the diverse body of literature on this topic and large range of study designs and methodologies before considering undertaking a systematic review. This scoping review further aimed to identify knowledge gaps and future research directions in the field of weight bias among exercise and fitness trainees and professionals.

## 2. Methods

**Figure 1:**
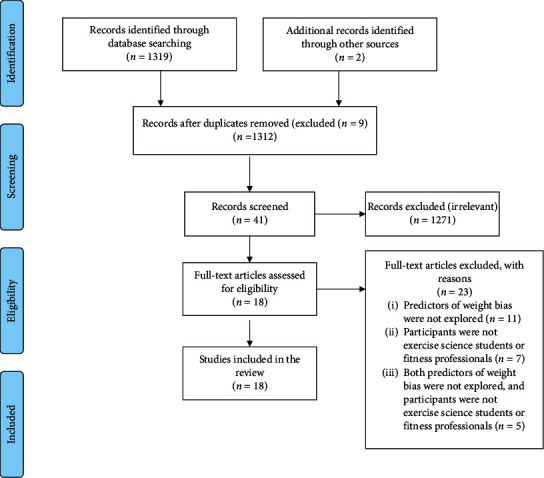
PRISMA-ScR flowchart illustrating the article selection process for the literature search.

We conducted a scoping review using the five-stage methodology outlined by Arksey and O'Malley [25]. With this method, all evidence and sources pertaining to our research question were gathered and summarized into overarching themes [25]. As such, scoping reviews are guided by a requirement to identify all relevant literature regardless of the heterogeneity of the body of literature, design, or quality [26]. This methodological approach is effective in presenting a broad overview of the literature on our research topic and is an effective way to identify research gaps [25]. The Preferred Reporting Items for Systematic reviews and Meta-Analyses extension for Scoping Reviews (PRISMA-ScR) flow diagram (see Figure 1) and checklist (Table 1) [27] were used to guide the reporting and show the steps taken in the article selection process of this review (see Appendix).

### 2.1. Literature Search

A literature search was designed and conducted in consultation with a health sciences librarian. PubMed and ERIC databases were searched on December 14, 2017, and again on May 8, 2019, using combinations of keywords and subject terms for weight, bias, health science students, and fitness professionals. Results were limited to articles published after January 1, 1990, and written in English or French. The complete search strategy for both databases can be found in the Appendix. Other articles (*n* = 2) were retrieved from the reference lists of pertinent studies that were identified in the personal libraries of the researchers.

### 2.2. Study Selection

Research studies that sought to understand the predictors and causes of weight bias in exercise science students and professionals were included in this scoping review. Only articles that included students and professionals in the fields of exercise science, physical education, kinesiology, physical therapy, fitness instruction, and exercise physiology were eligible for inclusion. Among selected articles, only those measuring the predictors or potential causes of weight bias in exercise science students and professionals were included for analysis. Studies that assessed weight bias in practicing health professionals including physicians, nurses, doctors, dietitians, psychologists, and social workers were not included in this review.

### 2.3. Data Charting

Reviewers (L.Z and T.S.) charted the characteristics of included studies in a table outlining title, authors, date of publication, country, study purpose, participant characteristics, methodology, and main findings. All authors verified the charted data for accuracy and the data are presented in Table 2.

## 3. Results

The literature search conducted on May 8, 2019, resulted in 1310 unique articles (after 9 duplicate articles were removed). An additional two articles were identified from the reference lists from the researchers' personal libraries resulting in a total of 1312 articles. These 1312 articles were screened and assessed for eligibility based on the inclusion criteria. Of the 41 articles that were screened as potentially relevant, 18 studies met the eligibility criteria and were included in the scoping review (Figure 1).

### 3.1. Characteristics of Included Studies

Table 2 shows the characteristics of included studies. More than half of the studies included were conducted in the USA (61.11%, *n* = 11) and used a combination of implicit and explicit measures to assess weight bias (61.11%, *n* = 11). The rest of the studies measured weight bias through explicit measures only (27.77%, *n* = 5) or through other measures (11.11%, *n* = 2) such as Q-methodology and one-on-one interviews to explore personal constructs of body shape and weight.

The majority of the studies (66.67%, *n* = 12) sampled undergraduate students enrolled in exercise science (*n* = 3), kinesiology (*n* = 3), physiology (*n* = 1), kinesiology, health promotion, and recreation (*n* = 1), health sciences (*n* = 1), and health and physical education training programs (*n* = 3). Four of the 18 studies (22.22%, *n* = 4) sampled fitness professionals only which included personal trainers and fitness instructors (*n* = 1), health educators (*n* = 1), fitness center employees (*n* = 1), and physical education professors (*n* = 1). Finally, one study (5.55%, *n* = 1) sampled both exercise science students and fitness professionals, e.g., physical education and exercise science students and athletes.

### 3.2. Themes

While conducting the thematic analysis according to Arksey and O'Malley [25], the researchers identified and grouped similar themes from each study' findings. Next, similar group themes were further synthesized into overarching themes. A total of six themes emerged to explain the predictors of weight bias in students and professionals in exercise science: beliefs in the controllability of weight; sex or gender differences; enrollment in a health sciences degree or program; psychosocial and personal factors; knowledge of obesity; lack of personal history, family, or friend with obesity.

### 3.3. Beliefs in the Personal Controllability of Weight

Eight studies [18, 21, 23, 33, 36–39] showed that exercise science students and professionals generally believe that weight is personally controllable. Common statements included “everyone has control over their weight” [39]; “eating right and exercising puts you on the right path for a long healthy life” [36]; and “physical activity is very important in the treatment of obesity” [33].

Exercise science students and professionals endorsing strong beliefs in weight controllability tended to explicitly associate people with obesity with “bad” attributes [21], “lazy” stereotypes and held higher explicit weight bias on social/character disparagement and weight control/blame attributes [18].

### 3.4. Sex Differences

Five studies reported sex or gender differences in the perception of weight toward individuals with obesity [18, 21, 29, 34, 35]. Two out of five studies found that men displayed higher weight bias attitudes than women [34, 35]. Indeed, according to Langdon et al., Exercise Science/Health students (ESHS) who were male tended to hold stronger explicit weight bias beliefs on weight control/blame, social/character disparagement, and physical/romantic subscales than ESHS who were female [34]. Alternatively, two studies found that women had stronger implicit weight bias toward individuals with obesity [18, 21] and were more likely to implicitly describe them as “bad” [18, 21] and “lazy” [21]. Two studies found no statistically significant differences in antifat bias scores between males and females [28, 29]. Although there is a relationship between sex and gender differences and weight bias, the studies have shown opposing findings in predicting the direction of the relationship with sex and gender.

### 3.5. Enrollment in a Health Sciences Degree or Program

Four studies compared weight bias in students majoring in health and exercise science to students enrolled in nonhealth disciplines, e.g., business, psychology, and other nonhealth majors [10, 17, 32]. Three studies showed that physical education and kinesiology students have higher weight bias compared to students enrolled in nonhealth degree programs [23]. O'Brien et al. showed that physical education students displayed higher levels of implicit and explicit weight bias as compared to psychology students [17]. Lynagh et al. found that enrollment in the health and physical education (HPE) specialist degree was a significant predictor of implicit weight bias and that HPE students held higher levels of weight bias compared to students in the nonspecialist teaching degree program [23]. Greenleaf et al. showed that students enrolled in Kinesiology, Health Promotion and Recreation (KHPR) had higher levels of explicit weight bias and endorsed stereotypes for children with obesity such as “likely to be teased”, “bad to be”, and “lazy” compared to non-KHPR majors [32]. However, one study by Robinson et al. found similar levels of both implicit and explicit weight bias in health science students enrolled in medicine, medical science, nursing/midwifery, pharmacy, dietetics, public health, exercise science, physiotherapy, etc., compared to nonhealth majors in business programs [10].

Interestingly, differences in levels of implicit weight bias were also found between year one and year three physical education students whereby third year students displayed higher levels of weight bias than first year physical education students [17, 19].

Enrollment in a health and exercise science program is a potential predictor of weight bias, although more research is needed to determine if weight bias increases in students enrolled in kinesiology or exercise science throughout the duration of their undergraduate degrees.

### 3.6. Psychosocial and Personal Factors

Six studies associated psychosocial factors, professional philosophies, and perceptions of self with weight bias in exercise science students and professionals [17, 29, 31, 33–35]. Psychosocial factors such as having ego-oriented goals and a tendency to internalize the athletic body ideal were measured in exercise science students. Students with ego-oriented goals “may avoid challenging tasks and feel discouraged when their performance is perceived as inferior to others” [34]. In this study, exercise science students were also likely to exhibit high internalization of the athletic body ideal and were “particularly susceptible to media messaging that idealizes the athletic body, portrayed as competent, competitive, and healthy” [34]. High internalization of the athletic body type ideal among exercise science students was found to be a predictor of fat phobia and weight control blame [34].

Social dominance orientation was cited as a predictor for explicit and implicit measures of weight bias whereby physical education students “see their own group as superior to and dominant over other relevant groups” [17]. Physical education students with high social dominance orientation displayed weight bias on explicit measures such as “dislike”, “fear of fat”, and “lack of willpower” and the implicit measures “bad” and “lazy” [17].

In fitness professionals, three studies have associated professional philosophies and perception of self with weight bias [17, 29, 35]. One study looked at different professional philosophies such as “behavior change”, “cognitive-based”, “decision-making”, “freeing/functioning”, and “social change” [29]. Among all professional philosophy measures, it was found that the “behavior change” education philosophy (i.e., emphasizing behavior modification as key in managing obesity) was associated with higher explicit weight bias in health educators [29]. In another study by Martinez-Lopez et al., 2010, self-efficacy expectations were measured to assess weight bias in physical education trainees toward youth with obesity. Self-efficacy in physical education trainees was defined as “the perceptions about their own capabilities to foster student's learning and engagement” [35]. Results showed that physical education trainees with higher levels of perceived self-efficacy displayed more favorable attitudes toward the educational treatment of children and youth obesity [35]. Another study sampled professors of physical education majors who consistently believed that “physical education teachers should not be obese, since they are role models for their students” [31]. A similar perception was also found in an earlier study conducted by Hare et al., 2000, whereby a sample of health fitness instructors, exercise test technologists, and exercise specialists believed that they should maintain normal weight to be role models for their clients/patients [33]. According to this study, most of the information on weight control was derived from textbooks, college courses, and scientific data [33].

### 3.7. Knowledge of Obesity

Three studies measured exercise science students' and professionals' knowledge of obesity in relation to weight bias [10, 19, 29]. One study examined perceived obesity education and found that health students who poorly rated their knowledge regarding the genetic causes of obesity had higher explicit weight bias on the “blame” subscale [10]. Kinesiology students enrolled in a nontraditional curriculum intervention emphasizing uncontrollable causes of weight (i.e., genetics) decreased explicit weight bias on the “blame” subscale compared to the control group of students who were learning the *traditional* curriculum focused on the role of exercise and diet in weight management [19]. One out of the three studies did not find a significant association between health educators' knowledge of obesity and weight bias [29].

### 3.8. Lack of Personal History, Family, or Friend with Obesity

A lack of personal history of overweight predicted high implicit weight bias measures of “bad” and “lazy” among a sample of fitness professionals and regular exercisers [21]. Chambliss et al., 2004, showed that a lack of family history of obesity and a lack of friends with obesity were associated with higher explicit weight bias [18]. However, DeBarr and Pettit reported that there were no statistical differences in weight bias between health educators who were overweight compared to their normal weight peers [29].

## 4. Discussion

In this scoping review, 18 studies were reviewed to identify predictors of weight bias among exercise science students and professionals. In the following section, we identify gaps from each of the six themes, discuss future research directions, and outline the strengths and limitations of this scoping review.

### 4.1. Future Research and Recommendations

This scoping review identified studies in undergraduate students in physical education, exercise science, and kinesiology and two studies assessed professionals in fitness instruction health education. To our knowledge, we could not find studies that assessed predictors of weight bias among practicing kinesiologists, physiotherapists, and athletic therapists although one study assessed a mixed sample of exercise professionals including sports physiologists [30]. Future research is also warranted to examine predictors of weight bias in other health sectors and settings (e.g., public health). While studies have shown that weight bias from primary care providers negatively affects quality of care and healthcare utilization of patients with obesity [11], impacts on the behaviors, treatment, and quality of care of individuals with obesity have yet to be assessed systematically in exercise science and physical education practice settings.

Only three studies measured knowledge of obesity in relation to weight bias in exercise science students and professionals in this scoping review [10, 19, 29]. To better understand exercise science students and professionals' behaviors toward overweight and obesity, future research should seek to examine the contents of exercise science course curricula that may foster and potentially sustain weight bias in exercise science students. Because it has been shown that weight bias increases in physical education students as they progress through their educational programs [17], it should also be determined if weight bias increases from the start to the completion of exercise science programs as well. Future weight bias reduction interventions should be designed to address these potential predictors of weight bias and evaluate their impact throughout students' educational training in exercise science programs.

Eight of the studies included in this scoping review identified beliefs of controllability of weight as a predictor of weight bias [18, 21, 23, 33, 36–39]. Crandall (1994) coined the phrase “ideology of blame” to define the dominant social belief that individuals are personally responsible for their weight. This social belief may explain exercise science students and professionals' weight bias. Studies also show that exposure to simulated courses emphasizing the controllable aspect of weight and the rigid concepts of “eating less and moving more” may lead to higher weight bias [40]. One study showed that exercise science students believed obesity to be preventable and controllable through diet restriction and energy expenditure highly valuing diet and exercise for weight management and weight loss [39]. The study suggested a lack of knowledge on other therapeutic interventions including bariatric surgery and that there is still resistance on understanding obesity as a complex condition [39]. This paper highlights the need to increase awareness of the complexity of obesity in the curriculum offered to exercise science students and more research to understand the causes of students' resistance to adopt and learn new concepts about obesity.

Few studies exist to explain sex differences in weight bias attitudes and have shown mixed results [18, 21, 29, 34, 35]. It is unclear how sex or gender plays a role in weight bias [34]. Although it has been proposed that women may be more sensitive to weight bias due to their higher vulnerability to the “thin ideal” [21, 41], further research studies should be designed to be adequately powered to examine potential sex and gender differences and in exercise science students and professionals. It would also be important to determine causes of differences in weight bias between sexes and genders in this field.

Other areas that warrant further study are the potential influences of ethnicity and setting on the development of weight bias (i.e., how are individuals with obesity seen when observed in a neutral setting versus being seen in an exercise facility/setting). Two articles showed mixed results in ethnicity as a potential predictor of weight bias in exercise science students [18, 28]; one stated no differences in explicit weight bias between American and Mexican athletes [28] while another found that exercise science students of Caucasian ethnicity living in a rural environment had higher levels of weight bias compared to those of other ethnicities [18]. With regard to setting, one study found that fitness center employees exhibited moderately strong implicit weight biases regardless of the setting in which they found themselves in (i.e., both neutral and exercise settings) [30]. The context in which the weight bias judgments were made did not affect the strength of implicit weight bias [30], suggesting weight bias still exists regardless of context in this aforementioned study. More research is needed to determine whether setting may act as a predictor of weight bias in fitness trainees and professionals.

One study by Fontana et al. evaluated the attitudes of professors in physical education departments toward individuals with obesity [31]. The sample of professors teaching physical education in this study held high implicit weight bias and disapproved physical education teachers with obesity as role models to students. This study demonstrated that greater explicit weight bias was associated with stronger disapproval of physical educators who have obesity as roles models and accepting physical education student majors living with obesity [31]. An earlier study by Hare et al. also showed that exercise professionals thought they should maintain normal weight to be role models for their clients [33]. These findings suggest a potential relationship between the importance exercise science students and professionals place on appearance and body weight (appearance orientation and body image preoccupation) with their current or future career as exercise science professionals. This suggests that more research is warranted on internalized weight bias (“the belief that negative stereotypes about weight apply to the self” [42]) in exercise science students and professionals to better understand underlying root causes of these internalized beliefs about weight, body image, and being role models in their field.

### 4.2. Strengths and Limitations

The present study is the first, to our knowledge, to gather the existing literature on predictors of weight bias in exercise science students and professionals. This scoping review provides a comprehensive summary of the overarching themes that emerged from the published studies that explored this topic. This comprehensive review helps identify predictors of weight bias that can serve as potential targets to address with curriculum changes and interventions aimed to improve training on the complexity of obesity and reduce weight bias in the early formative years before students become professionals in the field. However, since our scoping review focused on predictors of weight bias in exercise science students and professionals, suggestions for future research and interventions drawn from this paper can only be made about students and professionals in exercise science, kinesiology, and physical education fields.

## 5. Conclusion

This scoping review identified many overarching themes that predict weight bias in exercise science students and professionals. Belief in the personal controllability of weight was found to be the most researched and consistent predictor of weight bias in our population of interest. There appeared to be sex differences in weight bias that warrants further study; enrollment in a health sciences-related degree or program; psychosocial and personal factors relating to philosophy and personalities; traditional knowledge of obesity focusing mostly on diet and exercise; and lastly, a lack of personal history, family, or friend with obesity. Future research studies are needed to better understand predictors of weight bias in other health and exercise science-related fields, understand the impact of curricula that is heavily based on lifestyle factors only such as diet and exercise on weight bias, and evaluate the impact of weight bias reduction interventions in undergraduate students and professionals in the field of exercise science, kinesiology, and physical education.

## Figures and Tables

**Table 1:** Preferred Reporting Items for Systematic reviews and Meta-Analyses extension for Scoping Reviews (PRISMA-ScR) checklist.

Section	Item	PRISMA-ScR checklist ITEM	Reported on PAGE #
Title			
Title	1	Identify the report as a scoping review.	Page 1

Abstract			
Structured summary	2	Provide a structured summary that includes (as applicable): background, objectives, eligibility criteria, sources of evidence, charting methods, results, and conclusions that relate to the review questions and objectives.	Page 2

Introduction			
Rationale	3	Describe the rationale for the review in the context of what is already known. Explain why the review questions/objectives lend themselves to a scoping review approach.	Pages 3–5
Objectives	4	Provide an explicit statement of the questions and objectives being addressed with reference to their key elements (e.g., population or participants, concepts, and context) or other relevant key elements used to conceptualize the review questions and/or objectives.	Pages 4–6

Methods			
Protocol and registration	5	Indicate whether a review protocol exists; state if and where it can be accessed (e.g., a Web address); and if available, provide registration information, including the registration number.	Page 5
Eligibility criteria	6	Specify characteristics of the sources of evidence used as eligibility criteria (e.g., years considered, language, and publication status), and provide a rationale.	Pages 5–6
Information sources^*∗*^	7	Describe all information sources in the search (e.g., databases with dates of coverage and contact with authors to identify additional sources), as well as the date the most recent search was executed.	Pages 5
Search	8	Present the full electronic search strategy for at least 1 database, including any limits used, such that it could be repeated.	Pages 34–36
Selection of sources of evidence†	9	State the process for selecting sources of evidence (i.e., screening and eligibility) included in the scoping review.	Pages 5–6
Data charting process^‡^	10	Describe the methods of charting data from the included sources of evidence (e.g., calibrated forms or forms that have been tested by the team before their use, and whether data charting was done independently or in duplicate) and any processes for obtaining and confirming data from investigators.	Pages 6, 20–33
Data items	11	List and define all variables for which data were sought and any assumptions and simplifications made.	Pages 20–33
Critical appraisal of individual sources of evidence§	12	If done, provide a rationale for conducting a critical appraisal of included sources of evidence; describe the methods used and how this information was used in any data synthesis (if appropriate).	N/A
Synthesis of results	13	Describe the methods of handling and summarizing the data that were charted.	Pages 6, 20–33

Results			
Selection of sources of evidence	14	Give numbers of sources of evidence screened, assessed for eligibility, and included in the review, with reasons for exclusions at each stage, ideally using a flow diagram.	Page 19
Characteristics of sources of evidence	15	For each source of evidence, present characteristics for which data were charted and provide the citations.	Pages 20–33
Critical appraisal within sources of evidence	16	If done, present data on critical appraisal of included sources of evidence (see item 12).	N/A
Results of individual sources of evidence	17	For each included source of evidence, present the relevant data that were charted that relate to the review questions and objectives.	Pages 20–33
Synthesis of results	18	Summarize and/or present the charting results as they relate to the review questions and objectives.	Pages 20–33

Discussion			
Summary of evidence	19	Summarize the main results (including an overview of concepts, themes, and types of evidence available), link to the review questions and objectives, and consider the relevance to key groups.	Pages 6–11
Limitations	20	Discuss the limitations of the scoping review process.	Pages 14–15
Conclusions	21	Provide a general interpretation of the results with respect to the review questions and objectives, as well as potential implications and/or next steps.	Page 15

Funding			
Funding	22	Describe sources of funding for the included sources of evidence, as well as sources of funding for the scoping review. Describe the role of the funders of the scoping review.	Page 15

JBI = Joanna Briggs Institute; PRISMA-ScR = Preferred Reporting Items for Systematic reviews and Meta-Analyses extension for Scoping Reviews. ^*∗*^Where *sources of evidence* (see second footnote) are compiled from, such as bibliographic databases, social media platforms, and Web sites. ^†^A more inclusive/heterogeneous term used to account for the different types of evidence or data sources (e.g., quantitative and/or qualitative research, expert opinion, and policy documents) that may be eligible in a scoping review as opposed to only studies. This is not to be confused with *information sources* (see first footnote). ^‡^The frameworks by Arksey and O'Malley (6) and Levac and colleagues (7) and the JBI guidance (4, 5) refer to the process of data extraction in a scoping review as data charting. ^§^The process of systematically examining research evidence to assess its validity, results, and relevance before using it to inform a decision. This term is used for items 12 and 19 instead of “risk of bias” (which is more applicable to systematic reviews of interventions) to include and acknowledge the various sources of evidence that may be used in a scoping review (e.g., quantitative and/or qualitative research, expert opinion, and policy document).

**Table 2:** Characteristics of included studies (*n* = 18).

Ref #	Author	Year	Title	Geographical location	Study purpose	Academic background of participants	Sample description	Sample BMI classification	Study design	Weight bias measures used	Main findings
[28]	Alameda W. M., Whitehead R. J.	2015	Comparing levels of anti-fat bias between American and Mexican athletes and undergraduate physical education and exercise science students	United States of America	To study and compare explicit and implicit antifat bias ratings of Mexican and American samples of undergraduate physical education and exercise science (PEX) students and Mexican athletes. The study also aims to investigate psychometric concerns: the possibility that the explicit (AFAT) measure is prone to eliciting socially desirable (SD) response tendencies.	114 (45 women, 69 men) Mexican and American PEX students and Mexican athletes (*n* = 15) recruited through their university programs or coaches	Ages range from 18 to 65 yrs. The sample was predominantly of Caucasian and Hispanic ethnicity.	N/A	Descriptive statistics intercorrelations between AFAT and IAT subscales	The antifat attitudes test (AFAT) questionnaire, the implicit association test, balanced inventory of desirable responding questionnaire version 6 (BIDR-6) to account for the possibility that socially desirable response tendencies could affect the validity of explicit measures.	American and Mexican samples did not show problematic levels of explicit antifat bias, and both samples scored similarly on all three subscales (social/character disparagement, physical/romantic unattractiveness, weight control/blame) of the AFAT measure. Implicit bias results were different: The American sample scored higher in the good-bad and in the motivated-lazy subscales, but not the smart-stupid subscale. No significant gender differences in explicit/implicit weight bias.

[18]	Chambliss H.O., Finley C.E., and Blair S. N.	2004	Attitudes towards individuals with obesity among exercise science students	United States of America	To evaluate attitudes towards individuals with obesity and to identify personal characteristics associated with antifat bias among students majoring in exercise science.	Undergraduate (*N* = 136) and graduate students (*N* = 110)	136 undergraduate and 110 graduate exercise science students representing three colleges in Texas and Alabama. 55% of the participants are male, 77% of Caucasian origin with a mean age of 23.2 years.	N/A	Descriptive statistics and one-sample *t*-tests	Explicit and implicit measures: antifat attitudes test (AFAT) for explicit reports of weight bias and implicit association test (IAT) for automatic associations with obesity. Demographics questionnaire to correlate personal characteristics with weight bias.	Exercise science students demonstrated strong unconscious weight bias on the “bad” and “lazy” attributes. Women had stronger implicit bias on the good/bad measure but not on the lazy/motivated measure compared to men. Being Caucasian or growing up in a more rural environment was also associated with more negative attitudes on the good/bad measure.

[29]	DeBarr K., and Pettit M.	2016	Weight matters: health educators' knowledge of obesity and attitudes toward people who have obesity	United States of America	To assess health educators' professional philosophies, knowledge of obesity, and beliefs in a just world (meaning that people get what they deserve) in relation to their attitudes toward people who have obesity.	Self-identified health educators (*N* = 93). Nearly half (44.1%) of the participants held PhDs. Of the remaining, 22.6% held MA/MS degrees, 14% held BA/BS degrees, 10.8% held an MPH degree, and 8.6% indicated that their education degree could be described by specifying “other”	Self-identified health educators (*N* = 93) with an age range from 23 to 80 and a mean age of 45.48 (SD = 12.53). 73 participants were female and 20 were male.	2.2% classified as underweight, 68.8% normal, 28.0% had overweight, and 1.1% had obesity.	Cross-sectional study	(1) Welle et al.'s item to assess health educators' professional philosophies. (2) Items from the AFAT scale, global belief in a just world scale, and obesity knowledge scale. (3) Demographic items	Health educators who endorse the behavior modification philosophy or who have stronger beliefs in a just world (“people get what they deserve”) are more likely to hold antifat attitudes than those who believe that their role is one of providing assistance in a nonjudgmental fashion or engaging in political activism or advocacy.

[30]	Dimmock J. A., Hallett B. E, and Grove R.	2009	Attitudes toward overweight individuals among fitness center employees: an examination of contextual effects	Australia	To determine whether fitness center employees possess implicit and explicit weight bias and evaluate the extent of implicit bias in neutral versus exercise contexts.	70 employees (40 women, 30 men, mean age: 27 years) from various health and fitness centers in Australia	The sample included management and administrative staff (*n* = 15), personal trainers (*n* = 16), fitness instructors (*n* = 19), and exercise/sport physiologists (*n* = 20).	N/A	IAT algorithm	Antifat attitudes test (AFAT), two implicit association tests (IAT): 1. Participants responded to pictures of overweight and thin individuals in a neutral context. 2. Participants responded to pictures of the same individuals exercising on a treadmill.	Fitness center employees did not possess a strong explicit bias against individuals with overweight. Implicit ratings were moderate with ratings for weight control/blame being the highest, followed by the ratings for physical/romantic attractiveness.

[31]	Fontana F., Furtado O., Mazzardo O., Hong D., and de Campos W.	2017	Anti-fat bias by professors teaching physical education majors	United States of America	To evaluate the attitudes of professors in physical education departments towards individuals with obesity.	94 PETE (physical education teacher education) professors	94 PETE (physical education teacher education) professors from state universities across four US regions (northeast, midwest, south, and west).	N/A	One-sample *t*-tests were conducted to assess IAT and AFAS measures	Antifat attitude scale for measures of explicit negative attitudes, implicit association test for a measurement of implicit antifat bias.	PETE professors with higher levels of antifat bias more strongly opposed accepting majors who have obesity and disapproved of teachers who have obesity as role models to students. It is speculated that professors with higher levels of antifat bias strongly agreed that physical education teachers should maintain a normal body weight to set a good example to students.

[32]	Greenleaf C., Martin S. B., and Rhea D.	2008	Fighting fat: How do fat stereotypes influence beliefs about physical education?	United States of America	To examine college students' beliefs about youth obesity, the roles of schools and physical education in addressing obesity, and the training they receive to work with youth with overweight.	Physical education-related (*n* = 212) and nonphysical education-related (*n* = 218) majors	Male (*n* = 230) and female (*n* = 200) college students ranging from 18 to 45 years (mean age of 22.25 SD = 3.65).	8 individuals were considered underweight, 260 were normal weight, 114 were overweight, and 48 were obese according to the BMI classification.	2 (major) ×2 (fat stereotype group) design	Demographic questionnaire, modified fat stereotypes questionnaire, and perceptions of physical education questionnaire.	College students who endorsed antifat beliefs believe that future PE teachers should treat obesity in youth. They believe that maintaining their own weight will set a good example for youth with overweight.

[33]	Hare W. H., Price J. H., Flynn M. G., King K. A.	2000	Attitudes and perceptions of fitness professionals regarding obesity	United States of America	To evaluate the perceptions of exercise professionals toward obesity.	Certified health instructors, exercise test technologists, and exercise specialists certified by the American College of Sports Medicine (ACSM)	Range from 21 to 90 years.	N/A	ANOVA and ANCOVA analyses	Demographics questionnaire, 25-item questionnaire derived from Price's questionnaire (initially created to study medical professionals' perceptions on obesity).	The majority of the sample believed that physical activity is important in the treatment of obesity and that they should maintain a normal weight to be role models for their clients.

[34]	Langdon J., Rukavina P., and Greenleaf C.	2016	Predictors of obesity bias among exercise science students	United States of America	To investigate particular psychosocial predictors of obesity bias in prehealth professionals, which include the internalization of athletic and general body ideals, perceived media pressure and information, and achievement goal orientations.	Exercise science undergraduate students (*N* = 242)	Exercise science undergraduate students (*N* = 242), mean age of 20.93 years (SD = 1.78 years), 40% of the sample was men and 60% was women.	2.1% were underweight, 60% were normal weight, 28.8% had overweight, and 9.2% had obesity according to the BMI classification.	Questionnaire survey	AFAT and fat phobia scale to assess explicit weight bias. Sociocultural attitudes toward appearance. Questionnaire-3 (SATAQ-3) to assess media influences on body image. The modified task and ego sport questionnaire to assess personal goal orientations.	Students who displayed explicit obesity bias had higher task and ego goals, especially in varsity male students. Students with high weight bias also tended to internalize the athletic body ideal rather than the thin ideal as portrayed by the media.

[23]	Lynagh M., Cliff K., and Morgan P. J.	2015	Attitudes and beliefs of nonspecialist and specialist trainee health and physical education teachers toward obese children: evidence for “anti-fat” bias	Australia	To assess the beliefs and attitudes of preservice health and physical education (HPE) specialist and nonspecialist schoolteachers towards children with obesity.	Nonspecialist (*N* = 177) and HPE specialist (*N* = 62)	177 second-year preservice nonspecialist and 62 HPE specialist teaching students. 60% were between 18 and 20 years. Healthy BMI: 50%, overweight: 18%. Mean BMI: 23.6	Mean BMI or 23.6 (SD = 4.5).	Descriptive statistics, one-sample *t*-tests	Demographics questionnaire, the implicit association test (IAT), the 12-item antifat attitudes questionnaire (AFAQ), beliefs about causes of obesity in children: 1. 8-item beliefs about obese people scale (BAOP), 2. 12 items relating to factors believed to contribute to child obesity were included from the perceptions of youth obesity and physical education questionnaire (PYO and PEQ), the attitudes toward obese people scale (ATOP), expectations of overweight youth (EOY) questionnaire.	Enrollment in the HPE specialist degree was a significant predictor of both implicit bad/good antifat bias and implicit bias on the stupid/smart scale of the IAT. Most preservice teachers consider childhood obesity to be predominantly due to behavioral factors such as insufficient physical activity and sedentary lifestyle.

[35]	Martinez-Lopez E., Sanchez M. Z., Alvarez M. R., and Cruz M. T.	2010	Self-efficacy expectations in teacher trainees and the perceived role of schools and their physical education department in the educational treatment of overweight students	Spain	To study the relation between self-efficacy expectations and the attitude toward child and youth obesity, as well as the role of the school in this matter.	Specialist physical education trainees (*N* = 436)	Specialist physical education trainees (*N* = 436), mean age of 21.68 years (SD = 2.42) and ranged between 20 and 31 years. 292 participants were male and 144 were female.	86% participants considered themselves to be endomorphic, 251 mesomorphic, and 99 ectomorphic.	Multiple case study	Demographics questionnaire, physical activity questionnaire, a modified questionnaire based on Greenleaf and Weiller's (2005) “perceptions of youth obesity among physical educators” questionnaire, administered in Spanish.	Trainees who scored high on their perceived self-efficacy in the assessment of their own teaching methods and in the progress witnessed in students with overweight had more favorable attitudes toward these students.

[17]	O'Brien K. S., Hunter J. A., and Banks M.	2007	Implicit anti-fat bias in physical educators: physical attributes, ideology and socialization	New Zealand	To investigate the implicit and explicit prejudice of physical education (HPE) students before and following extensive professional training and to examine the relationship of antifat prejudice to relevant psychosocial predictors.	344 university students	180 physical education students and 164 psychology students, 67% female, mean age of 20 years	Mean BMI of 23.18 kg/m^2^_._	2 × 2 factorial design	Implicit association test (IAT), Crandall's 13-item antifat attitudes questionnaire for explicit measures. Investment in physical attributes: 1. Two items were constructed to assess the importance of mathematical and physical abilities 2. Luhtanen and Crocker's four-item identity scale to assess how central a specific identity is to one's self-concept (“being a PE student is an important reflection of who I am”), the body esteem scale, the 14-item unidimensional SDO scale.	PE students displayed higher levels of implicit antifat bias than psychology students and other health professionals. Additionally, year three PE students displayed higher levels of implicit antifat attitudes than year one PE students. The higher implicit antifat biases exhibited by year three PE students were associated with SDO and lower body esteem.

[36]	Richardson L. A., Fister C. L., and Ramlo S. E.	2015	Effect of an exercise and weight control curriculum: views of obesity among exercise science students	United States of America	To investigate student views of weight management and obesity based on course content featuring current views of weight management, weight loss, and bariatric care.	Undergraduate exercise science students (*N* = 22)	22 junior and senior-level exercise science students enrolled in the exercise and weight control course. Gender division: 8 men and 14 women. Mean age of 24.5 ± 6.68 yrs.	N/A	Pretest/posttest Q-methodology	Precourse and postcourse Q sorts were compared in exercise students before and after exposure to the exercise and weight course.	Following the course, “assimilator learners” showed more acceptance of obesity condition in accordance with comprehensive course content. “Askew learners” did not accept course content due to their own focus remaining on diet restrictions and energy expenditure while believing that obesity is completely preventable and controllable.

[21]	Robertson N., and Vohora R.	2008	Fitness vs. fatness: implicit bias toward obesity among fitness professionals and regular exercisers	United Kingdom	To assess implicit attitudes toward obesity among two key groups of people in a public exercise setting: fitness professionals offering exercise advice and regular exercisers.	Fitness professionals (*N* = 57) and regular exercisers/undergraduate students (*N* = 56)	Fitness professionals (*N* = 57) (gym instructors *n* = 54, aerobics instructors *n* = 3). 32 males and 25 females with a mean age of 29–30 years (SD = 10.23). Regular exercisers (*N* = 56). 22 males and 34 females with a mean age of 20.67 (SD = 2.41).	Fitness professionals mean BMI of 24.09 (SD = 4.77), regular exercisers mean BMI of 22.08 (SD = 3.14).	Questionnaire survey, within-subjects design	Demographic questionnaire, semantic differential measure of explicit beliefs, and the implicit associations test (IAT).	Evidence of a strong antifat bias was found for both fitness professionals and regular exercisers on all implicit and explicit measures (good vs. bad; motivated vs. lazy). This bias was more pronounced for fitness professionals who themselves had never been overweight and who believed personal control dictated body weight. For regular exercisers, a higher level of antifat bias was found for females, younger participants, and those who had never been overweight.

[10]	Robinson E. L., Ball L. E., and Leveritt M. D.	2014	Obesity bias among health and non-health students attending an Australian university and their perceived obesity education	Australia	To compare the level of prejudice against individuals with obesity (obesity bias) among final-year health and nonhealth students.	479 final-year students (292 health and 187 nonhealth)	Mean age of 26.2 ± 7.6 years.	Mean BMI of 23.2 ± 4.7 kg/m^2^_._	Cross-sectional study	IAT antifat attitudes questionnaire (AFAQ).	Both health and nonhealth students displayed weight bias. Nonhealth students attributed obesity to personal lack of willpower. Students who believed obesity was genetics-related had more favorable views.

[37]	Rukavina P. B., Li W., and Rowell M. B.	2008	A service learning based intervention to change attitudes towards individuals with obesity in kinesiology pre-professionals	United States of America	To conduct an intervention to change attitudes towards individuals with obesity.	Kinesiology undergraduate students (*N* = 95)	69 participants from 4 different undergraduate concentrations (38 clinical exercise physiology, 10 fitness management, 13 physical education preservice teachers, and 8 sport communication majors).	Mean BMI of 25.52 (SD = 4.45).	Quantitative and qualitative test design	Demographics questionnaire explicit ratings test (ERT), antifat attitudes test (AFAT).	The kinesiology preprofessionals did not report high overall towards individuals with obesity. However, certain antifat attitudes and stereotypes were endorsed in areas of weight control/blame and physical/romantic attractiveness but not in their character.

[38]	Rukavina P. B., Li W., Shen B., and Sun H.	2010	A service learning based project to change implicit and explicit bias towards individuals with obesity in kinesiology pre-professionals	United States of America	To assess the efficacy of a multicomponent intervention to reduce kinesiology preprofessionals' implicit and explicit bias.	Kinesiology preprofessionals (*N* = 78)	78 kinesiology preprofessionals (51 male and 26 females, mean age of 21.63 years SD = 1.49 years)	N/A	Pre-post experimental design with a control group	Demographics questionnaire explicit ratings test (ERT), antifat attitudes test (AFAT), and implicit association test (IAT).	On the pretest, participants did not display overall explicit bias on the antifat attitudes test but had strong implicit bias on the stupid/smart and lazy/motivated semantic differential scale. Preprofessionals also endorsed individual stereotypes related to individual control of lifestyle behavior and outward appearance. Participation in the intervention reduced explicit bias on the AFAT social character disparagement and weight control/blame subscales but not implicit bias.

[39]	Varea V., and Underwood M.	2016	“You are just an idiot for not doing any physical activity right now”: pre-service health and physical education teachers' constructions of fatness	Australia	To explore how a group of preservice health and physical education (HPE) specialist teachers from an Australian university construct fatness discourses.	Students in their second year of HPE training (*N* = 14)	14 students (11 females and 3 males) between the ages of 18 and 26 years.	N/A	Qualitative semistructured interviews	One-on-one interviews to explore personal constructs and discourses related to body, health, and fatness photo elicitation during interview provide a reflective essay after interview.	Weight attitudes: “Fat is an indicator of health and fitness”, “fat is an achieved deviance” (fat individuals can be considered “achieved deviants”, as they earn their deviant status on the strength due to lack of self-control). Most of the participants constructed discourses associated with fat bodies being disgusting or repulsive.

[19]	Wijayatunga N. N., Kim Y. Butsch W. S., and Dhurandhar E. J.	2019	The effects of a teaching intervention on weight bias among kinesiology undergraduate students	United States of America	To test if learning about uncontrollable causes of obesity and the causes of weight bias would reduce explicit and implicit weight bias among kinesiology undergraduate students compared to the traditional curriculum focused on controllable causes of weight gain.	Undergraduate students majoring in kinesiology and sport management (*N* = 67)	Study participants have a mean age of 21.76 (SD = 1.43).	Mean BMI of 25.09 (SD = 4.51 kg/m^2^).	Prospective, quasi-experimental study	A sociodemographic questionnaire, antifat attitude test (AFAT), and IAT were administered at preintervention, immediately after completion of the two-day in-class intervention, and one month after the intervention.	A teaching intervention that highlights the uncontrollable causes of obesity (unlike the traditional curriculum that emphasizes controllable causes of obesity) reduced weight blame component of explicit weight bias in kinesiology major students. Learning about diet and exercise interventions to treat obesity appears to increase implicit weight bias in kinesiology students.

## Data Availability

The data supporting this scoping review are from previously reported studies and datasets, which have been cited. The processed data are available from the corresponding author upon request.

## Appendix

Database search strategy 2017 and 2019 is as follows:

1. Anti-fat[tiab] OR “anti fat”[tiab] OR “fat phobia”[tiab] OR “fat phobic”[tiab]
2. “Body Mass Index”[Mesh] OR “Body Weight”[Mesh] OR “obesity”[Mesh] OR “overweight”[Mesh] OR obese[tiab] OR obesity[tiab] OR overweight[tiab] OR “overweight”[tiab] OR weight[tiab] OR “Obesity/psychology”[Mesh]
3. “Bias (Epidemiology)”[Mesh] OR “prejudice”[Mesh] OR “Social Stigma”[Mesh] OR “stereotyping”[MeSH] OR bias[tiab] OR biased[tiab] OR biases[tiab] OR discriminate[tiab] OR discriminates[tiab] OR discriminated[tiab] OR discrimination[tiab] OR prejudice[tiab] OR prejudiced[tiab] OR stereotype[tiab] OR stereotypes[tiab] OR stereotyped[tiab] OR stereotyping[tiab] OR stigma[tiab] OR stigmas[tiab] OR stigmatization[tiab] OR stigmatize[tiab] OR stigmatized[tiab] OR stigmatizes[tiab] OR stigmatizing[tiab] OR stigmatisation[tiab] OR stigmatise[tiab] OR stigmatised[tiab] OR stigmatises[tiab] OR stigmatising[tiab] OR empathy[tiab] OR trust[tiab] OR “Negative interaction”[tiab] OR “negative encounter”[tiab] OR “negative experience”[tiab] OR shame[tiab] OR shaming[tiab] OR shamed[tiab] OR “Attitude to Health”[Mesh] OR “Attitude of Health Personnel”[Mesh] OR “Health Knowledge, Attitudes, Practice”[Mesh] OR “Prejudice”[Mesh]
4. #1 OR (#2 AND #3)
5. “Physical Education and Training”[Mesh] OR “Health Occupations/education”[Mesh] OR “Students, Health Occupations/psychology”[Mesh]
6. “Health science”[tiab] OR “health sciences”[tiab] OR “exercise science”[tiab] OR “exercise sciences”[tiab] OR “kinesiology”[tiab] OR “physiology”[tiab] OR “physical education”[tiab]
7. “Pre-professionals”[tiab] OR “prehealth professional”[tiab] OR “non-specialist”[tiab] OR “trainee”[tiab ] OR “student”[tiab] OR “students”[tiab]
8. #5 OR (#6 AND #7)
9. “Physical Fitness/psychology”[Mesh] OR “Fitness Centers/manpower”[Mesh]
10. “Fitness”[tiab] OR “kinesiologists”[tiab] OR “kinesiologist”[tiab] OR “physical education”[tiab] OR “kinesiology”[tiab] OR “physiology”[tiab] OR “Health professional”[tiab] OR “Health professionals”[tiab]
11. Specialist[tiab] OR trainer[tiab] OR employee[tiab] OR professional[tiab] OR professionals[tiab] OR specialists[tiab] OR employees[tiab] OR teacher[tiab] OR teachers[tiab] OR instructor[tiab] OR instructors[tiab]
12. #9 OR (#10 AND #11)
13. #4 AND (#8 OR #12)

Filters:
    Date: 1990/01/01 to current (Search conducted December 14, 2017 and updated May 8, 2019)
    Language: English OR French

Search conducted in Eric on EBSCOhost on December 14, 2017 and updated May 8, 2019.

1. (weight OR fat OR anti-fat OR obesity OR obese OR Overweight) AND (bias OR stigma OR negative attitude OR Prejudice)
2. (Educator OR Teacher OR Professional OR Personnel OR professors OR instructors OR students) AND (health OR fitness OR physical education OR kinesiology OR exercise science OR allied Health OR physiology)
3. (Athletic therapist OR Physiologists OR kinesiologists OR athlete OR pre-service teacher)
4. S1 AND (S2 OR S3)
